# Supplementary material for: Splice-Junction-Based Mapping of Alternative Isoforms in the Human Proteome
Source: Cell Rep. Author manuscript; Available in PMC 2020 Jan 15. (PMC6961840; doi:10.1016/j.celrep.2019.11.026)
Supplement: 2 [file NIHMS1546469-supplement-2.zip › DF1/PXD010154/Heart/Heart_47_MYBPC3_MPEPGKKPEKAEPMLAPAPAPAEATGAPGEAPAPAAELGESAPSPK.pdf]

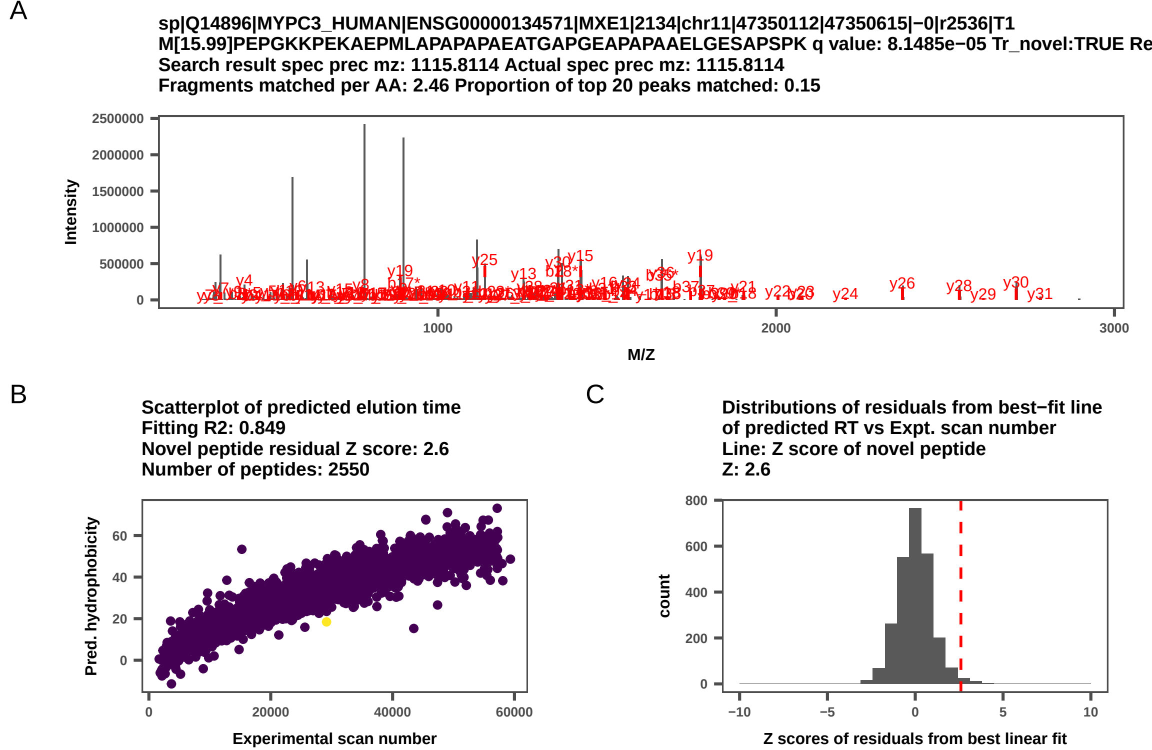
A
sp|Q14896|MYPC3_HUMAN|ENSG00000134571|MXE1|2134|chr11|47350112|47350615|−0|r2536|T1
M[15.99]PEPGKKPEKAEPMLAPAPAPAEATGAPGEAPAPAAELGESAPSPK q value: 8.1485e−05 Tr_novel:TRUE Re
Search result spec prec mz: 1115.8114 Actual spec prec mz: 1115.8114
Fragments matched per AA: 2.46 Proportion of top 20 peaks matched: 0.15
Intensity
M/Z
B
Scatterplot of predicted elution time
Fitting R2: 0.849
Novel peptide residual Z score: 2.6
Number of peptides: 2550
Pred. hydrophobicity
Experimental scan number
C
Distributions of residuals from best−fit line
of predicted RT vs Expt. scan number
Line: Z score of novel peptide
Z: 2.6
count
Z scores of residuals from best linear fit
